# Dimethyl 8-acetyl-2-methyl-1,2-dihydro­quinoline-2,4-dicarboxyl­ate

**DOI:** 10.1107/S1600536811003564

**Published:** 2011-02-02

**Authors:** Zeynep Keleşoğlu, Zeynep Gültekin, Orhan Büyükgüngör

**Affiliations:** a Department of Physics, Ondokuz Mayıs University, TR-55139 Samsun, Turkey; b Department of Chemistry, Çankırı Karatekin University, TR-18100 Çankırı, Turkey

## Abstract

In the title compound, C_16_H_17_NO_5_, the six-membered N-containing ring has a half-boat form; the spiro C atom deviates by 0.34 (2) Å from the plane (r.m.s. deviation = 0.051 Å) defined by the N and four aromatic C atoms. Intra­molecular N—H⋯O hydrogen bonding generates an *S*(6) ring motif and the dihedral angle between the mean plane though the *S*(6) ring and that through the five-atom half-boat plane is 3.39 (2)°. In the crystal, weak inter­molecular C—H⋯O hydrogen bonds link mol­ecules into zigzag chains along [001] due to *c*-glide symmetry, and C—H⋯π inter­actions extend along [010].

## Related literature

For the preparation of 1,2-dihydro­quinoline, see: Dauphinee & Forrest (1978 ▶); Katritzky *et al.* (1996 ▶); Elmore *et al.* (2001 ▶); Lu & Malinakova (2004 ▶); Wang *et al.* (2009 ▶); Rezgui *et al.* (1999 ▶). For related structures, see: Yadav *et al.* (2007 ▶); Kamakshi & Reddy (2007 ▶); Kim *et al.* (2001 ▶); Sundèn *et al.* (2007 ▶); Waldmann *et al.* (2008 ▶). For ring puckering analysis, see: Cremer & Pople (1975 ▶). For graph-set theory, see: Bernstein *et al.* (1995 ▶).
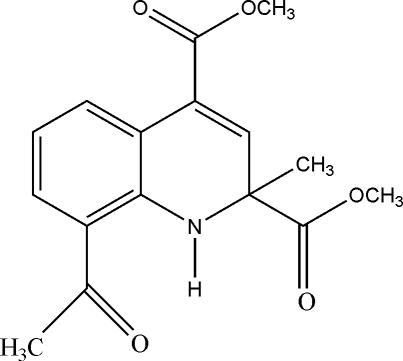


## Experimental

### 

#### Crystal data

C_16_H_17_NO_5_
*M* *_r_* = 303.31
Monoclinic, 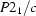

*a* = 8.0222 (3) Å
*b* = 18.2466 (9) Å
*c* = 10.3478 (4) Å
β = 101.042 (3)°
*V* = 1486.65 (11) Å^3^
*Z* = 4
Mo *K*α radiation
μ = 0.10 mm^−1^
*T* = 296 K
0.74 × 0.43 × 0.23 mm

#### Data collection

Stoe IPDS 2 diffractometer
Absorption correction: integration (*X-RED32*; Stoe & Cie, 2002 ▶) *T*_min_ = 0.823, *T*_max_ = 0.968
14613 measured reflections
3068 independent reflections
2221 reflections with *I* > 2σ(*I*)
*R*_int_ = 0.055

#### Refinement

*R*[*F* ^2^ > 2σ(*F* ^2^)] = 0.059
*wR*(*F* ^2^) = 0.169
*S* = 1.07
14613 reflections
205 parameters
1 restraint
H atoms treated by a mixture of independent and constrained refinement
Δρ_max_ = 0.43 e Å^−3^
Δρ_min_ = −0.36 e Å^−3^

### 

Data collection: *X-AREA* (Stoe & Cie, 2002 ▶); cell refinement: *X-AREA*; data reduction: *X-RED32* (Stoe & Cie, 2002 ▶); program(s) used to solve structure: *SHELXS97* (Sheldrick, 2008 ▶); program(s) used to refine structure: *SHELXL97* (Sheldrick, 2008 ▶); molecular graphics: *ORTEP-3 for Windows* (Farrugia, 1997 ▶); software used to prepare material for publication: *WinGX* (Farrugia, 1999 ▶).

## Supplementary Material

Crystal structure: contains datablocks I, global. DOI: 10.1107/S1600536811003564/si2329sup1.cif

Structure factors: contains datablocks I. DOI: 10.1107/S1600536811003564/si2329Isup2.hkl

Additional supplementary materials:  crystallographic information; 3D view; checkCIF report

## Figures and Tables

**Table 1:** Hydrogen-bond geometry (Å, °) *Cg*1 is the centroid of the C1–C6 ring.

*D*—H⋯*A*	*D*—H	H⋯*A*	*D*⋯*A*	*D*—H⋯*A*
N1—H1⋯O1	0.86 (2)	1.95 (2)	2.650 (2)	138 (2)
C10—H10⋯O3^i^	0.93	2.59	3.517 (3)	174
C14—H14*C*⋯*Cg*1^i^	0.96	2.89	3.692 (3)	142
C8—H8*B*⋯*Cg*1^ii^	0.96	2.85	3.501 (3)	126

Symmetry codes: (i) 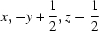; (ii) 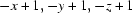.

**Fig. 1.**
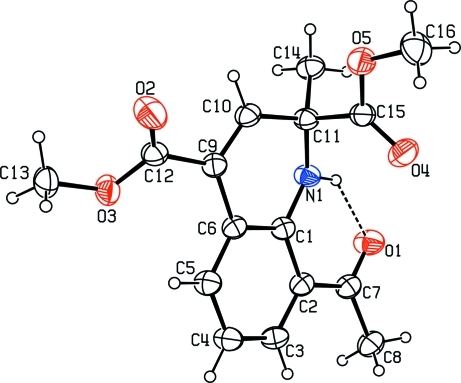
An ORTEP view of (I), with the atom-numbering scheme and 30% probability displacement ellipsoids. Dashed lines indicate H-bonds.

**Fig. 2.**
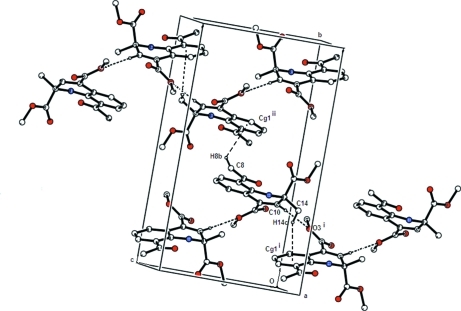
A packing diagram for (I), showing the C—H···O hydrogen bonds and C—H···π interactions. H atoms not involved in hydrogen bonding (dashed lines) have been omitted for clarity. [Symmetry code; (i): x,1/2 - y,-1/2 + z; (ii): 1 - x,1 - y,1 - z]. (Cg1 is the centroid of the C1—C6 ring).

## supplementary crystallographic information

### Comment

Dihydroquinoline moiety is found in a wide variety of natural products and they have attracted a lot of attention from synthetic organic chemists (Kamakshi & Reddy, 2007). 1,2-Dihydroquinolines have received substantial attention due to their potential biological activities arising from their antioxidative properties as well as their usefulness as precursors of some other biologically active compounds (Kim *et al.*, 2001). 1,2-Dihydroquinoline derivatives are known to exhibit a wide spectrum of biological activities such as antimalarial, antibacterial and anti-inflammatory behavior (Yadav *et al.*, 2007).

Asymmetric synthesis of 1,2-dihydroquinolin have been attracted in recent years (Wang *et al.*, 2009; Rezgui *et al.*, 1999). Sundèn and co-worker has reported asymmetric synthesis of 1,2-dihydroquinolines using Domino reactions between 2-aminobenzaldehyde and α,β-unsaturated aldehydes to give pharmaceutically valuable 1,2-dihydroquinolines in high enantioselectivity (Sundèn *et al.*, 2007). 1,2-dihydroquinolines was used for several applications, such as synthesis of quinolines: (Dauphinee & Forrest, 1978; Lu & Malinakova, 2004), 1,2,3,4- tetrahydroquinolines: (Katritzky *et al.*, 1996) and natural products: (Elmore *et al.*, 2001).

The molecule of the title compound contains dihydroquinoline, two methoxycarbonyl (O=C—O—CH_3_) and acetyl group (O=C—CH_3_). The two –CO_2_Me groups are antisymmetric with respect to atom C10 (Fig. 1).

The six-membered N containing ring of the quinoline system displays a half-boat conformation with the puckering parameters of Q_T_=0.261 (2) Å, Φ=146.7 (6)° and Θ=112.7 (4) ° (Cremer & Pople, 1975) and the spiro carbon atom deviates 0.34 (2) Å from the plane (r.m.s. deviation 0.051 Å) defined by the N1 and C1, C6, C9, C10 atoms. The intramolecular N—H..O hydrogen bond generate S(6) ring motif (Bernstein *et al.*, 1995) (Fig. 1, Table 1). The dihedral angle between the S(6) ring mean plane and the half-boat plane of five atoms is 3.39 (2)°.

The crystal packing is stabilized by C10—H10···O3^i^ intermolecular hydrogen bonds linking the molecules into chains in a zigzag mode along [0 0 1] due to *c*-glide symmetry, and there are also two C—H···π interactions C14—H14*c*···*Cg*1^i^ and C8—H8b···*Cg*1^ii^ [(i): *x*,1/2 - *y*,-1/2 + *z*; (ii):1 - *x*,1 - *y*,1 - *z*] extending along the *b* axis (Fig. 2., Table 1.).

### Experimental

The title compound was synthesized after a method described by Waldmann *et al.*, (2008). 2'-aminoacetophenone (100 mg, 1 eq) was dissolved in acetonitrile (1.5 ml) in a screw-capped test tube and Bi(OTf)_3_ (5 mol %, 0.05 eq) was added to the mixture. This mixture were stirred at room temperature for 4 days until the starting material was completely consumed as monitored by TLC. The resultant residue was directly purified by flash chromatography on silica (EtOAc: Cyclohexane 2:98) gave 27% yield as a yellow solid. Recrystallized over pentan and ethyl acetate gave yellow crystalline solid. *R*_f_ 0.5 (2:1 Cyclohexane/EtOAc); m.p: (374–375 K).

### Refinement

The H atom of the NH group was located in a difference Fourier map and refined with the constraint N—H = 0.86 (2) Å. All other H atoms were positioned with idealized geometry using a riding model, [C—H = 0.93–0.96Å and *U*_iso_ = 1.2*U*_eq_(C)].

### Crystal data

**Table:**

C_16_H_17_NO_5_	*F*(000) = 640
*M**_r_* = 303.31	*D*_x_ = 1.355 Mg m^−^^3^
Monoclinic, *P*2_1_/*c*	Mo *K*α radiation, λ = 0.71073 Å
Hall symbol: -P 2ybc	Cell parameters from 14613 reflections
*a* = 8.0222 (3) Å	θ = 2.0–28.0°
*b* = 18.2466 (9) Å	µ = 0.10 mm^−^^1^
*c* = 10.3478 (4) Å	*T* = 296 K
β = 101.042 (3)°	Prism, brown
*V* = 1486.65 (11) Å^3^	0.74 × 0.43 × 0.23 mm
*Z* = 4	

### Data collection

**Table:**

Stoe IPDS 2 diffractometer	3068 independent reflections
Radiation source: fine-focus sealed tube	2221 reflections with *I* > 2σ(*I*)
graphite	*R*_int_ = 0.055
rotation method scans	θ_max_ = 26.5°, θ_min_ = 2.2°
Absorption correction: integration (*X-RED32*; Stoe & Cie, 2002)	*h* = −10→10
*T*_min_ = 0.823, *T*_max_ = 0.968	*k* = −22→22
14613 measured reflections	*l* = −12→12

### Refinement

**Table:**

Refinement on *F*^2^	Primary atom site location: structure-invariant direct methods
Least-squares matrix: full	Secondary atom site location: difference Fourier map
*R*[*F*^2^ > 2σ(*F*^2^)] = 0.059	Hydrogen site location: inferred from neighbouring sites
*wR*(*F*^2^) = 0.169	H atoms treated by a mixture of independent and constrained refinement
*S* = 1.07	*w* = 1/[σ^2^(*F*_o_^2^) + (0.0846*P*)^2^ + 0.2827*P*] where *P* = (*F*_o_^2^ + 2*F*_c_^2^)/3
14613 reflections	(Δ/σ)_max_ < 0.001
205 parameters	Δρ_max_ = 0.43 e Å^−^^3^
1 restraint	Δρ_min_ = −0.36 e Å^−^^3^

### Special details

**Table:**

Geometry. All e.s.d.'s (except the e.s.d. in the dihedral angle between two l.s. planes) are estimated using the full covariance matrix. The cell e.s.d.'s are taken into account individually in the estimation of e.s.d.'s in distances, angles and torsion angles; correlations between e.s.d.'s in cell parameters are only used when they are defined by crystal symmetry. An approximate (isotropic) treatment of cell e.s.d.'s is used for estimating e.s.d.'s involving l.s. planes.
Refinement. Refinement of *F*^2^ against ALL reflections. The weighted *R*-factor *wR* and goodness of fit *S* are based on *F*^2^, conventional *R*-factors *R* are based on *F*, with *F* set to zero for negative *F*^2^. The threshold expression of *F*^2^ > σ(*F*^2^) is used only for calculating *R*-factors(gt) *etc*. and is not relevant to the choice of reflections for refinement. *R*-factors based on *F*^2^ are statistically about twice as large as those based on *F*, and *R*- factors based on ALL data will be even larger.

### Fractional atomic coordinates and isotropic or equivalent isotropic displacement parameters (Å^2^)

**Table:**

	*x*	*y*	*z*	*U*_iso_*/*U*_eq_	
C1	0.4104 (2)	0.37012 (12)	0.3615 (2)	0.0427 (5)	
C2	0.5203 (3)	0.39441 (12)	0.4782 (2)	0.0458 (5)	
C3	0.4559 (3)	0.39964 (14)	0.5939 (2)	0.0537 (6)	
H3	0.5267	0.4161	0.6702	0.064*	
C4	0.2911 (3)	0.38117 (16)	0.5987 (2)	0.0582 (6)	
H4	0.2512	0.3853	0.6771	0.070*	
C5	0.1846 (3)	0.35628 (15)	0.4853 (2)	0.0529 (6)	
H5	0.0732	0.3436	0.4887	0.064*	
C6	0.2403 (2)	0.34998 (13)	0.3675 (2)	0.0436 (5)	
C7	0.6980 (3)	0.41517 (14)	0.4779 (2)	0.0507 (6)	
C8	0.8065 (3)	0.44802 (16)	0.5984 (3)	0.0618 (7)	
H8A	0.8402	0.4104	0.6630	0.074*	
H8B	0.7431	0.4850	0.6341	0.074*	
H8C	0.9058	0.4697	0.5751	0.074*	
C9	0.1341 (2)	0.32351 (13)	0.2442 (2)	0.0454 (5)	
C10	0.1863 (3)	0.32944 (14)	0.1302 (2)	0.0491 (5)	
H10	0.1169	0.3113	0.0547	0.059*	
C11	0.3514 (3)	0.36395 (13)	0.1178 (2)	0.0462 (5)	
C12	−0.0356 (3)	0.28883 (15)	0.2385 (2)	0.0521 (6)	
C13	−0.2120 (3)	0.21400 (18)	0.3394 (3)	0.0732 (8)	
H13A	−0.2844	0.2489	0.3708	0.088*	
H13B	−0.1979	0.1719	0.3960	0.088*	
H13C	−0.2625	0.1993	0.2515	0.088*	
C14	0.4362 (3)	0.32316 (16)	0.0183 (2)	0.0572 (6)	
H14A	0.5410	0.3470	0.0119	0.069*	
H14B	0.3620	0.3234	−0.0663	0.069*	
H14C	0.4588	0.2735	0.0469	0.069*	
C15	0.3165 (3)	0.44329 (14)	0.0688 (2)	0.0496 (6)	
C16	0.1661 (4)	0.51590 (18)	−0.1039 (3)	0.0788 (9)	
H16A	0.1176	0.5463	−0.0450	0.095*	
H16B	0.0861	0.5102	−0.1851	0.095*	
H16C	0.2678	0.5384	−0.1211	0.095*	
N1	0.4639 (2)	0.36371 (12)	0.24524 (18)	0.0508 (5)	
O1	0.7624 (2)	0.40668 (13)	0.38037 (19)	0.0708 (6)	
O2	−0.1528 (2)	0.29932 (15)	0.1485 (2)	0.0906 (8)	
O3	−0.0469 (2)	0.24724 (11)	0.33918 (18)	0.0657 (5)	
O4	0.3796 (3)	0.49676 (12)	0.1243 (2)	0.0794 (6)	
O5	0.2062 (2)	0.44499 (10)	−0.04465 (18)	0.0641 (5)	
H1	0.566 (2)	0.3785 (15)	0.248 (3)	0.065 (8)*	

### Atomic displacement parameters (Å^2^)

**Table:**

	*U*^11^	*U*^22^	*U*^33^	*U*^12^	*U*^13^	*U*^23^
C1	0.0390 (10)	0.0448 (12)	0.0419 (11)	0.0046 (9)	0.0017 (8)	0.0007 (9)
C2	0.0438 (11)	0.0449 (12)	0.0450 (12)	0.0039 (9)	−0.0014 (9)	0.0003 (10)
C3	0.0524 (12)	0.0619 (16)	0.0425 (12)	−0.0001 (11)	−0.0015 (10)	−0.0073 (11)
C4	0.0540 (13)	0.0755 (18)	0.0454 (13)	0.0000 (12)	0.0099 (10)	−0.0068 (12)
C5	0.0463 (11)	0.0635 (15)	0.0486 (13)	0.0016 (10)	0.0081 (10)	−0.0037 (11)
C6	0.0397 (10)	0.0455 (12)	0.0431 (12)	0.0025 (8)	0.0018 (8)	−0.0009 (9)
C7	0.0441 (11)	0.0536 (14)	0.0507 (14)	0.0021 (10)	−0.0005 (10)	−0.0018 (11)
C8	0.0503 (12)	0.0684 (17)	0.0611 (16)	−0.0086 (11)	−0.0031 (11)	−0.0069 (13)
C9	0.0392 (10)	0.0497 (13)	0.0446 (12)	0.0008 (9)	0.0013 (9)	0.0021 (10)
C10	0.0422 (10)	0.0575 (14)	0.0440 (12)	−0.0040 (10)	−0.0007 (9)	−0.0034 (10)
C11	0.0407 (10)	0.0574 (14)	0.0391 (11)	−0.0018 (9)	0.0041 (8)	−0.0030 (10)
C12	0.0461 (12)	0.0657 (16)	0.0424 (12)	−0.0063 (10)	0.0027 (10)	−0.0011 (11)
C13	0.0639 (15)	0.086 (2)	0.0689 (18)	−0.0292 (15)	0.0113 (13)	0.0083 (16)
C14	0.0509 (12)	0.0676 (17)	0.0534 (15)	0.0019 (11)	0.0108 (10)	−0.0106 (12)
C15	0.0463 (11)	0.0599 (15)	0.0428 (12)	−0.0056 (10)	0.0095 (9)	−0.0046 (11)
C16	0.0801 (19)	0.077 (2)	0.074 (2)	0.0029 (16)	0.0012 (15)	0.0221 (17)
N1	0.0375 (9)	0.0726 (14)	0.0405 (10)	−0.0042 (9)	0.0032 (8)	−0.0029 (9)
O1	0.0446 (9)	0.1052 (16)	0.0605 (11)	−0.0100 (9)	0.0051 (8)	−0.0129 (11)
O2	0.0528 (10)	0.149 (2)	0.0624 (12)	−0.0263 (11)	−0.0078 (9)	0.0270 (13)
O3	0.0573 (9)	0.0731 (13)	0.0615 (11)	−0.0174 (8)	−0.0020 (8)	0.0178 (10)
O4	0.1003 (15)	0.0624 (12)	0.0673 (13)	−0.0137 (11)	−0.0043 (11)	−0.0087 (10)
O5	0.0648 (10)	0.0631 (12)	0.0566 (11)	−0.0039 (8)	−0.0079 (8)	0.0080 (9)

### Geometric parameters (Å, °)

**Table:**

C1—N1	1.358 (3)	C10—H10	0.9300
C1—C2	1.423 (3)	C11—N1	1.448 (3)
C1—C6	1.426 (3)	C11—C14	1.531 (3)
C2—C3	1.395 (3)	C11—C15	1.542 (3)
C2—C7	1.475 (3)	C12—O2	1.205 (3)
C3—C4	1.374 (3)	C12—O3	1.306 (3)
C3—H3	0.9300	C13—O3	1.457 (3)
C4—C5	1.389 (3)	C13—H13A	0.9600
C4—H4	0.9300	C13—H13B	0.9600
C5—C6	1.381 (3)	C13—H13C	0.9600
C5—H5	0.9300	C14—H14A	0.9600
C6—C9	1.474 (3)	C14—H14B	0.9600
C7—O1	1.229 (3)	C14—H14C	0.9600
C7—C8	1.502 (3)	C15—O4	1.195 (3)
C8—H8A	0.9600	C15—O5	1.328 (3)
C8—H8B	0.9600	C16—O5	1.442 (3)
C8—H8C	0.9600	C16—H16A	0.9600
C9—C10	1.330 (3)	C16—H16B	0.9600
C9—C12	1.492 (3)	C16—H16C	0.9600
C10—C11	1.494 (3)	N1—H1	0.858 (17)
			
N1—C1—C2	121.97 (18)	N1—C11—C14	109.33 (18)
N1—C1—C6	118.91 (19)	C10—C11—C14	111.6 (2)
C2—C1—C6	119.11 (19)	N1—C11—C15	110.15 (19)
C3—C2—C1	118.56 (19)	C10—C11—C15	108.43 (18)
C3—C2—C7	120.1 (2)	C14—C11—C15	108.15 (19)
C1—C2—C7	121.3 (2)	O2—C12—O3	123.1 (2)
C4—C3—C2	122.1 (2)	O2—C12—C9	122.3 (2)
C4—C3—H3	118.9	O3—C12—C9	114.68 (19)
C2—C3—H3	118.9	O3—C13—H13A	109.5
C3—C4—C5	119.3 (2)	O3—C13—H13B	109.5
C3—C4—H4	120.3	H13A—C13—H13B	109.5
C5—C4—H4	120.3	O3—C13—H13C	109.5
C6—C5—C4	121.5 (2)	H13A—C13—H13C	109.5
C6—C5—H5	119.3	H13B—C13—H13C	109.5
C4—C5—H5	119.3	C11—C14—H14A	109.5
C5—C6—C1	119.4 (2)	C11—C14—H14B	109.5
C5—C6—C9	124.05 (19)	H14A—C14—H14B	109.5
C1—C6—C9	116.55 (19)	C11—C14—H14C	109.5
O1—C7—C2	121.9 (2)	H14A—C14—H14C	109.5
O1—C7—C8	117.6 (2)	H14B—C14—H14C	109.5
C2—C7—C8	120.5 (2)	O4—C15—O5	123.7 (2)
C7—C8—H8A	109.5	O4—C15—C11	125.1 (2)
C7—C8—H8B	109.5	O5—C15—C11	111.1 (2)
H8A—C8—H8B	109.5	O5—C16—H16A	109.5
C7—C8—H8C	109.5	O5—C16—H16B	109.5
H8A—C8—H8C	109.5	H16A—C16—H16B	109.5
H8B—C8—H8C	109.5	O5—C16—H16C	109.5
C10—C9—C6	120.85 (19)	H16A—C16—H16C	109.5
C10—C9—C12	116.1 (2)	H16B—C16—H16C	109.5
C6—C9—C12	123.04 (19)	C1—N1—C11	124.00 (17)
C9—C10—C11	123.1 (2)	C1—N1—H1	114.2 (19)
C9—C10—H10	118.5	C11—N1—H1	116.7 (19)
C11—C10—H10	118.5	C12—O3—C13	116.44 (19)
N1—C11—C10	109.17 (18)	C15—O5—C16	117.0 (2)
			
N1—C1—C2—C3	179.9 (2)	C12—C9—C10—C11	178.9 (2)
C6—C1—C2—C3	−1.8 (3)	C9—C10—C11—N1	20.8 (3)
N1—C1—C2—C7	1.1 (3)	C9—C10—C11—C14	141.8 (2)
C6—C1—C2—C7	179.3 (2)	C9—C10—C11—C15	−99.2 (3)
C1—C2—C3—C4	0.9 (4)	C10—C9—C12—O2	−37.6 (4)
C7—C2—C3—C4	179.8 (2)	C6—C9—C12—O2	142.5 (3)
C2—C3—C4—C5	0.2 (4)	C10—C9—C12—O3	143.2 (2)
C3—C4—C5—C6	−0.3 (4)	C6—C9—C12—O3	−36.7 (3)
C4—C5—C6—C1	−0.6 (4)	N1—C11—C15—O4	3.5 (3)
C4—C5—C6—C9	179.6 (2)	C10—C11—C15—O4	122.9 (3)
N1—C1—C6—C5	180.0 (2)	C14—C11—C15—O4	−115.9 (3)
C2—C1—C6—C5	1.7 (3)	N1—C11—C15—O5	−176.59 (17)
N1—C1—C6—C9	−0.2 (3)	C10—C11—C15—O5	−57.2 (2)
C2—C1—C6—C9	−178.5 (2)	C14—C11—C15—O5	64.0 (2)
C3—C2—C7—O1	175.0 (3)	C2—C1—N1—C11	−158.1 (2)
C1—C2—C7—O1	−6.1 (4)	C6—C1—N1—C11	23.6 (3)
C3—C2—C7—C8	−5.2 (4)	C10—C11—N1—C1	−32.7 (3)
C1—C2—C7—C8	173.6 (2)	C14—C11—N1—C1	−155.0 (2)
C5—C6—C9—C10	169.4 (2)	C15—C11—N1—C1	86.3 (3)
C1—C6—C9—C10	−10.4 (3)	O2—C12—O3—C13	−0.9 (4)
C5—C6—C9—C12	−10.7 (4)	C9—C12—O3—C13	178.3 (2)
C1—C6—C9—C12	169.5 (2)	O4—C15—O5—C16	1.8 (4)
C6—C9—C10—C11	−1.3 (4)	C11—C15—O5—C16	−178.1 (2)

### Hydrogen-bond geometry (Å, °)

**Table:**

Cg1 is the centroid of the C1–C6 ring.

**Table:**

*D*—H···*A*	*D*—H	H···*A*	*D*···*A*	*D*—H···*A*
N1—H1···O1	0.86 (2)	1.95 (2)	2.650 (2)	138 (2)
C10—H10···O3^i^	0.93	2.59	3.517 (3)	174
C14—H14C···Cg1^i^	0.96	2.89	3.692 (3)	142
C8—H8B···Cg1^ii^	0.96	2.85	3.501 (3)	126

Symmetry codes: (i) *x*, −*y*+1/2, *z*−1/2; (ii) −*x*+1, −*y*+1, −*z*+1.
